# Impaired brain glucose metabolism in glucagon-like peptide-1 receptor knockout mice

**DOI:** 10.1038/s41387-024-00343-w

**Published:** 2024-10-10

**Authors:** Hui Li, Yujiao Fang, Da Wang, Bowen Shi, Garth J. Thompson

**Affiliations:** 1 iHuman Institute, ShanghaiTech University, Shanghai, China; 2 School of Life Science and Technology, ShanghaiTech University, Shanghai, China

**Keywords:** Diseases of the nervous system, Metabolism

## Abstract

**Background:**

Quantitative mapping of the brain’s metabolism is a critical tool in studying and diagnosing many conditions, from obesity to neurodegenerative diseases. In particular, noninvasive approaches are urgently required. Recently, there have been promising drug development approaches for the treatment of disorders related to glucose metabolism in the brain and, therefore, against obesity-associated diseases. One of the most important drug targets to emerge has been the Glucagon-like peptide-1 (GLP-1) and its receptor (GLP-1R). GLP and GLP-1R play an important role in regulating blood sugar and maintaining energy homeostasis. However, the macroscopic effects on brain metabolism and function due to the presence of GLP-1R are unclear.

**Methods:**

To explore the physiological role of GLP-1R in mouse brain glucose metabolism, and its relationship to brain function, we used three methods. We used deuterium magnetic resonance spectroscopy (DMRS) to provide quantitative information about metabolic flux, fluorodeoxyglucose positron emission tomography (FDG-PET) to measure brain glucose metabolism, and resting state-functional MRI (rs-fMRI) to measure brain functional connectivity. We used these methods in both mice with complete GLP-1R knockout (GLP-1R KO) and wild-type C57BL/6N (WT) mice.

**Results:**

The metabolic rate of GLP-1R KO mice was significantly slower than that of WT mice (*p* = 0.0345, WT mice 0.02335 ± 0.057 mM/min, GLP-1R KO mice 0.01998 ± 0.07 mM/min). Quantification of the mean [^18^F]FDG signal in the whole brain also showed significantly reduced glucose uptake in GLP-1R KO mice versus control mice (*p* = 0.0314). Observing rs-fMRI, the functional brain connectivity in GLP-1R KO mice was significantly lower than that in the WT group (*p* = 0.0032 for gFCD, *p* = 0.0002 for whole-brain correlation, *p* < 0.0001 for ALFF).

**Conclusions:**

GLP-1R KO mice exhibit impaired brain glucose metabolism to high doses of exogenous glucose, and they also have reduced functional connectivity. This suggests that the GLP-1R KO mouse model may serve as a model for correlated metabolic and functional connectivity loss.

## Introduction

The glucagon-like peptide-1 receptor (GLP-1R), is a prototypical class B GPCR involved in the regulation of glucose homeostasis, food intake, inflammation and hypertension [1, 2]. GLP-1R is widely expressed in the whole body, mainly in the lungs, kidneys, heart, adipose tissue, and the central and peripheral nervous systems [3]. GLP-1, the endogenous agonist of GLP-1R, is predominantly synthesized in enteroendocrine cells of the distal small intestine and secreted in the blood when food enters the duodenum [4, 5]. GLP-1 is a promising natural antidiabetic product due to its anorexigenic, insulinotropic, and weight-reducing effects [6].

The importance of GLP-1 and GLP-1R in blood glucose modulation has been fully recognized, mainly focused on the pancreas and intestinal system [7–9]. Recently, GLP-1 and GLP-1R signaling in the central nervous system has also been shown to regulate energy balance and glucose homeostasis. Rodent studies revealed that administration of GLP-1R agonists to the central or peripheral nervous system can activate a subset of GLP–1R–expressing neurons and produce weight loss [10, 11]. Gejl et al. demonstrated that the activation of GLP-1R affected blood-brain barrier transport and metabolism of glucose in the brain [12]. Sisley et al. used Cre-lox technology to delete the gene encoding the GLP-1R specifically in the CNS to reveal that GLP-1R agonist-induced weight loss requires GLP-1Rs in the CNS [13]. Therefore, GLP-1R is considered a potential therapeutic target for diseases related to brain glucose metabolic disorders.

Metabolism plays a key role in the origin or progression of many diseases, including neurodegenerative diseases, diabetes, and cancer [14]. Some metabolically sensitive imaging modalities can be used for clinical diagnosis, facilitating early detection and treatment of diseases. [^18^F]-fluorodeoxyglucose (FDG)-positron emission tomography (PET) can be considered the gold standard for metabolic imaging in the clinic. [^18^F] FDG-PET provides relatively high-resolution maps of glucose uptake, but it requires the use of a radioactive agent and it is incapable of providing mitochondrial oxidative metabolic information, due to it only measuring the ^18^FDG uptake in the cytosol. FDG-PET has been widely used for the diagnosis of neuronal disorders, such as Alzheimer’s Disease [15–17], brain tumors [18], and epilepsy [19, 20].

Magnetic resonance spectroscopy (MRS) is a noninvasive, non-radioactive method of imaging metabolism [21]. Proton magnetic resonance spectroscopy (^1^H MRS), with its extremely high sensitivity, is used for detecting and quantifying several endogenous tissue metabolites in a single acquisition, but it is not capable of tracking the activity of a metabolic pathway due to limitations related to water, lipid suppression, and magnetic field homogeneity [22]. Here, we have used a novel ^2^H MRS method, referred to as deuterium MRS (DMRS) [23], which has the potential for broad application in in vivo studies to assess brain metabolism and activity in preclinical and clinical settings. DMRS uses a wide range of deuterated substrates (including glucose and acetate) as markers, monitors their metabolites, and maps dynamic metabolism with high spatial/temporal resolution [24]. Due to its low natural abundance and short longitudinal relaxation time, the deuterium atom has a “clean” spectral background and has good magnetic resonance sensitivity. Hence, DMRS has strong potential and feasibility for in vivo metabolic imaging.

While PET provides high-sensitivity measurement of tissue glucose uptake with good spatial resolution, it does not provide any further information about downstream glucose metabolism. Thus, FDG-PET and DMRS are complementary in exploring the brain glucose metabolism of GLP-1R KO mice versus WT mice.

Resting-state functional magnetic resonance image (rs-fMRI) is an imaging technique based on the blood oxygen level-dependent (BOLD) signal, which measures changes in blood oxygen levels across the brain to infer functional activity [25]. It can also measure the spontaneous signal of networks within the brain during a resting state. Due to its good spatial and temporal resolution and relative ease of recording, it is widely used as a biomarker of disease and as a tool to measure the brain’s activity to monitor the effectiveness of disease treatment [26].

In this work, we investigated the physiological role of GLP-1R in mouse brain glucose metabolism. We used DMRS and FDG-PET to record the metabolic dynamics, and rs-fMRI to record the functional activity, from the brains of GLP-1R KO and WT mice. This is the first time that either DMRS or combined FDG-PET and rs-fMRI have been used in GLP-1R-related studies, as previous studies on GLP-1R have mainly focused on changes in pancreatic and peripheral blood glucose. We are also interested in the correlated changes across all three modalities, as the comparison between brain metabolism and function has important implications for medical imaging biomarkers, both when metabolism and function are correlated [27] and also when they diverge [28].

## Materials and methods

A diagram of the data collection procedures is shown in Fig. S1.

### Animals

All animal experiments were approved by the Institutional Animal Care and Use Committee of ShanghaiTech University and were consistent with the governmental regulations of China for the care and use of animals.

GLP-1R knockout mice (GLP-1R KO, Shanghai Model Organisms, based on C57BL/6N) were generated using CRISPR/Cas9 technology, which causes *Glp1r* gene protein reading frame shift and loss of function by using non-homologous recombination repair to introduce mutations. Briefly, the process is as follows: Cas9 mRNA and gRNA were obtained by in vitro transcription; Cas9 mRNA and gRNA were microinjected into the fertilized eggs of WT mice to obtain F0 generation mice. 11 positive F1 generation mice were obtained by mating the F0 generation mice identified as positive by PCR amplification and sequencing with C57BL/6N mice. Mutant mice were genotyped to ensure the deletion of the target *Glp1r* segment.

Male WT mice and male GLP-1R knockout mice, aged 7–13 weeks with weighing 20–30 g were included in this study [29]. Animals were housed under a 12 h light/dark cycle at a room temperature of 22 ± 1 °C with 45% humidity, given ad libitum access to food and water.

Based on existing literature, an N of 6 was initially tested for DMRS in both groups and the fMRI group of GLP-1R KO mice, an N of 7 for PET in both groups [30–33], and an N of 6 for both groups for cyclic adenosine monophosphate (cAMP) measurement. For the fMRI group of WT mice, we used data from 11 WT mice recorded with an identical protocol from a previous, unpublished study. As detailed in later sections, the DMRS spectra of one WT mouse was excluded due to failure in data processing, the fMRI data from one GLP-1R KO mouse was excluded due to being an outlier, and one GLP-1R KO mouse died during PET image acquisition. Therefore, the total number of mice used herein is 52. Five WT mice and six GLP-1R KO mice were imaged with DMRS. Seven WT mice and six GLP-1R KO mice were imaged with [^18^F]-FDG-PET. Eleven WT mice and Five GLP-1R KO mice were imaged with rs-fMRI. Six WT mice and six GLP-1R KO mice had cAMP measured. As results were already strongly statistically significant with these initial values, we did not add additional mice to the study under the principle of reduction in animal experiments [34].

Animals were not used for multiple experiments as two of the three experiments were non-survival (due to the blood collection following DMRS and the urethane anesthesia used for fMRI) and incompatible (DMRS and PET both required labeled glucose injection, the DMRS and fMRI signals may interfere with each other, and fMRI required different anesthesia than the other two experiments).

### Deuterium magnetic resonance spectroscopy

WT and GLP-1R KO mice were anesthetized by inhaling a mixture of 1–3% isoflurane and compressed air (1.5 L/min). The animals were not fasted prior to scanning. A 30 gauge needle and 70-cm-long venous catheter preflushed with a heparin-solution (50 IU/ml) solution was inserted into a lateral tail vein and fixed with adhesive tape to prevent blood clotting. During the entire experimental procedure, the animals were maintained under normal physiological conditions. Rectal temperature was maintained at 37 ± 1 °C using heated circulating water and a hot-air blower, and respiratory rate was monitored at ~80–120 breaths per minute.

DMRS experiments were performed in a 9.4 Tesla (BioSpec 90/30 USR; Bruker Biospin MRI, Ettlingen, Germany), using a custom-built ^1^H/^2^H birdcage radiofrequency (RF) coil (Larmor Device, Hangzhou, China) [35] as receiver and transmitter. The magnet was equipped with B-GA12SHP for BC70/20 gradient coil, and B-GA12SHP INSERT for BGA10SHP BC94/30 shimming coil. The ^1^H loop was tuned to 400.3 MHz for localizing the mouse brain and shimming, while the ^2^H loop was tuned to 61.4 MHz for acquiring ^2^H spectra. A deuterium water phantom (physiological saline solution containing 10% D_2_O) was used to calibrate the reference power at 60 W. In animal experiments, the coil center was positioned over the mouse brain. The 1_localizer sequence and ^1^H signal were employed for brain localization, and the 2_localizer_shim sequence was utilized for field homogenization. ^2^H-NMR spectra were obtained with the ^2^H loop and a single-pulse sequence, using saturation bands to suppress signals from tissues other than the brain. A single-pulse sequence was applied to acquire dynamic DMRS spectra from the mouse brain with the following parameters: 2 KHz bandwidth, 512 data points for the free induction decay (FID), 64° flip angle, 350 ms repetition time (TR) with 800 averages (5 min per spectrum) and a total of 15 spectra. For each mouse, baseline ^2^H spectra were obtained followed by around 1 min intravenous infusion of [6,6’]-^2^H_2_ glucose (d66, Aladdin; 1.95 g/kg, 1 M/L) and ~75 min continuous DMRS scanning.

For the first three mice, immediately following DMRS data collection, we took blood from the hearts to perform in vitro 2H-NMR with an 18.8 T nuclear magnetic resonance facility (800 MHz NMR) to validate our results recorded at a lower spectral resolution.

Mice were excluded if no peaks other than water could be distinguished from their spectrum. This resulted in the exclusion of one WT mouse’s data.

GLX and Lactate peaks were only visible in spectra for one WT mouse. An example of a spectrum from a single mouse with all four peaks visible is shown in Fig. S2. Therefore, our analysis focused on HDO and deuterium glucose. ^2^H resonance signal integrals (HDO and deuterium glucose) were fitted using MestReNova software (version 14.2.0, MestreLab Research, Spain) and MATLAB code for quantification. The ^2^H-NMR spectra were generated by 10 Hz exponential window function transformation, followed by zero-filling to generate 1024 FID data points (twice the original). Manual phase correction and multi-point baseline correction were performed, and the relevant steps were saved for batch processing. For ^2^H-NMR spectra, using deuterated water (HDO) as the chemical shift reference (~4.7 ppm), the peaks corresponding to d-glucose-6,6’-d2 were marked (around 3.8 ppm). These two signal peaks were fitted, and the area under the curve was extracted. According to known relaxation times from previous studies [23, 32], relaxation-corrected glucose concentrations were measured in dynamic DMRS. The decay equation for glucose is given in Eq. 1.

1 $$f\left(t\right)=Y0+\left(Y1-Y0\right)* {e}^{-{kt}}$$

In Eq. 1, $f$ represents concentration, $t$ represents time, $e$ is the base of natural logarithms, $Y0$ is the initial concentration of post-injection, $Y1$ is the final concentration, and $k$ is the rate constant, expressed in inverse minutes. The rate constant of brain glucose metabolism for each mouse was determined, and statistical analysis was performed on the two groups of samples using a Student’s *t*-test.

### Positron emission tomography

Mice were fasted for 12 h before imaging. 2-deoxy-2-[fluorine-18] fluoro-d-glucose ([18 F]-FDG) (Dongcheng AMS Pharmaceutical atom, Shanghai, China) was injected into the tail vein at a dose of ~3.7 MBq/10 g body weight (0.1 mCi/10 g). [^18^F]-FDG was distributed in the mice for 40 min while awake. Mice were imaged using a Bruker BioSpec 9.4 T scanner with PET insert (Bruker, Ettingen, Germany) for [^18^F]-FDG-PET imaging. To prevent movement contamination from confounding the result with ^18^F-FDG uptake in muscles, isoflurane anesthesia (5% for induction and ≤2% for maintenance) was used here for biodistribution detection and imaging. First, a T_2_-weighted MRI (T2WI) rapid acquisition with relaxation enhancement (RARE) (echo time (TE) = 54.39 ms, repetition time (TR) = 1800 ms, field of view (FOV) = 35 × 35 × 85 mm^3^, matrix size = 128 × 128) was performed. Then, the mice were imaged with 10-min static PET scanning. PET images were reconstructed with the ordered-subsets expectation maximization 2D algorithm (OSEM2D) in Paravision 360. PMOD v4.4 Software (Bruker, Ettingen, Germany) was used for image registration and quantitative analysis. Standardized uptake value (SUV) was calculated [SUV = radioactivity activity concentration (KBq/ml)/body weight (g)/injected dose (kBq)] for semi-quantitative analysis. Afterward, SUV values were corrected for body weight [SUV_bw_ = SUV × body weight (g/ml)].

### Resting-state functional MRI (rs-fMRI)

Functional MRI data were recorded using a Bruker BioSpec 9.4 T scanner, equipped with an 86-mm volume coil for transmitting, and a 4-channel phased array cryogenic mouse head coil (Bruker, Ettingen, Germany) for receiving. Animals were anesthetized with 25% urethane dissolved in distilled water (Sigma, U2500–100 G) using intraperitoneal bolus injections (1.3 g/kg) divided into three separate doses. The interval time was 10 min between each dose [36–38]. No ventilation is needed to maintain the hemodynamic state of animals under urethane. Sufficient anesthesia was judged by no toe-pinch reflex or weak toe-pinch response. A built-in warm water heating pad (Bruker) was used to maintain body temperature between 36 and 37.5 °C. Respiratory rates were monitored throughout, and respiration was maintained within the range of 180–220 breaths per minute.

Anatomical images were acquired using a T_2_-Turbo-RARE scan, TR = 2230 ms, TE = 39.43 ms, FOV = 20 × 7.9 mm^2^, slices: 23, slice thickness = 0.3 mm, matrix size = 200 × 79. A gradient-echo EPI sequence, TR = 1000 ms, TE = 13 ms, FOV = 20 × 7.9 mm^2^, slices: 13, slice thickness = 0.6 mm, matrix size = 121 × 48, repetitions = 480, was used to obtain resting state functional MRI (rs-fMRI) blood oxygen level-dependent (BOLD) data.

rs-fMRI BOLD data were preprocessed and analyzed using custom-written scripts in MATLAB. Motion correction and slice timing were performed in SPM12 (Wellcome Trust Centre for Neuroimaging, London, UK). Anatomical images were registered to the mouse brain template [39], using a nonlinear registration (50 iterations, normalized mutual information, and otherwise default) with BioImage Suite (Yale School of Medicine, 2015; bioimagesuite.yale.edu). Each mouse’s corresponding nonlinear registration was then applied to rs-fMRI data from the same mouse. To facilitate inter-mice statistics, each rs-fMRI image was spatially smoothed (0.5 mm isotropic Gaussian kernel), and each voxel’s time series was bandpass filtered from 0.01 to 0.3 Hz [40, 41].

### Per-voxel rs-fMRI metrics

The global signal is the average of the BOLD signal from all voxels, at each time point. Correlating the global signal with the time series of each voxel (Pearson’s correlation) yields a whole-brain correlation, which is also considered a “weighted” form of functional connectivity density [42]. A normalizing version of Fisher’s transformation was applied to normalize the Pearson correlation to a hypothetical N (0,1) distribution of Z scores [43]. Results were averaged together for all voxels within the whole brain.

The amplitude of low-frequency fluctuations (ALFF) in fMRI is a widely used metric for assessing spontaneous neuronal activity [44]. For each voxel, the power spectrum was computed from the fMRI time series, and then the square root of the power spectrum was obtained. The ALFF value was derived by averaging the square root values within the frequency range of 0.01–0.1 Hz [45]. Results were averaged together for all voxels within the whole brain.

Functional connectivity density (FCD) mapping, a metric derived from graph theory and employing rs-fMRI data, utilizes Pearson’s correlation to generate a map of functional connections in the brain [46]. Global functional connectivity density (gFCD) measures connections across the entire brain. For each voxel, we used a correlation threshold of *r* ≥ 0.25 to determine the number of voxels across the entire brain linked to it. The resulting gFCD values were calculated on a per-mouse basis. Results are averaged together for all voxels within the whole brain.

The global signal, ALFF, and FCD were tested in GraphPad (version 9.5.1 GraphPad Software, LLC) using the built-in ROUT method to find outliers. One GLP-1R KO mouse had outliers in all three metrics, and was thus excluded.

### cAMP measurement

cAMP levels in whole brain tissues were measured using a cAMP assay kit (Shanghai Boke Biotechnology Co., Ltd., Shanghai, China). Each mouse was euthanized, and their entire brain was removed and liquified to use as a single sample. Data from all mice (*N* = 6 per group) were used. The assay was performed according to the manufacturer’s instructions provided in the kit.

### Statistical analysis

We analyzed the distribution of these data and applied the F-test to test if the variances of the two populations were equal. Statistical analysis of the data was performed using a Student’s *t*-test. Statistical significance was set at *p* < 0.05. Effect size was used to quantify the differences between group means and the relationships between variables [47, 48]. The effect size was calculated here using the method of Cohen’s *d* for a *t*-test [49]. For non-normal distribution data, instead of standard deviation, we used the 84.13% percentile minus the median when we calculated the effect size [49]. Normally, from this calculation, effect sizes greater than 0.5 are considered a “large difference effect”. Detailed information regarding the testing for a normal distribution, *F*-test and statistical analysis results, and effect size results are summarized in Table S1.

## Results

### Decreased glucose metabolism in GLP-1R KO mice detected with DMRS

**Fig. 1:**
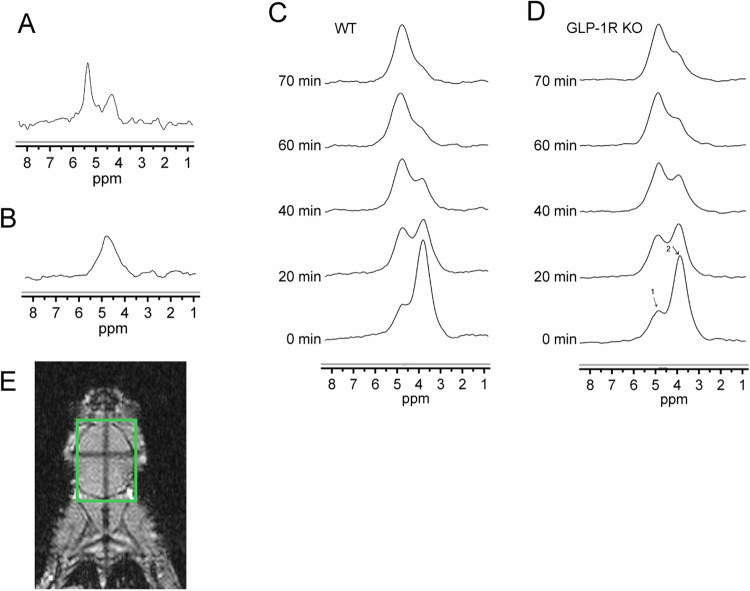
Metabolic dynamics measured with DMRS. **A** Water phantom containing 2.5 mM/L deuterated glucose. **B** Natural abundance of water in the brain prior to glucose injection. Average DMRS spectra from WT mice (**C**) and GLP-1R KO mice (**D**) after injection of [6,6]-^2^H_2_ glucose. Signal showed at 0, 20, 40, 60, and 70 min, in this order. First scan after glucose injection, set as 0 min. Each spectrum was acquired over ~5 min. (1) HDO, 4.7 ppm; (2) Glucose, 3.8 ppm. *N* = 6 for GLP-1R KO mice, *N* = 5 for WT mice. **E** The region of interest in the brain is highlighted in the green box.

We first tracked the dynamic changes of deuterium substrates in the brains of living mice with DMRS. Figure 1A shows the chemical shift of the phantom containing 2.5 mM/L deuterated glucose, which was used as a reference. Figure 1B shows the natural abundance of HDO in the mouse brain before injection of [6,6’]-^2^H_2_ glucose, which was used as an internal concentration reference. The natural abundance of deuterium is 0.0115%, and the water content of the mouse brain is ~80% at 55.5 M, so the HDO baseline of the mice brain is ~10.12 mM [23]. We scanned mice immediately after [6,6’]-^2^H_2_ glucose injection. Figure 1C, D shows the average spectra obtained from WT and GLP-1R KO mice, respectively, at the time points of 0, 20, 40, 60, and 70 min after the first scan, in this order. These spectra show the dynamic changes of both deuterated glucose and water in the mouse brain. We can see that after [6,6’]-^2^H_2_ glucose injection, the concentration of HDO increased gradually, while the concentration of glucose reached a maximum immediately after injection and then rapidly decreased. Figure 1E showed the region of interest (green box) where the mouse brain was not suppressed by saturation during DMRS.

**Fig. 2:**
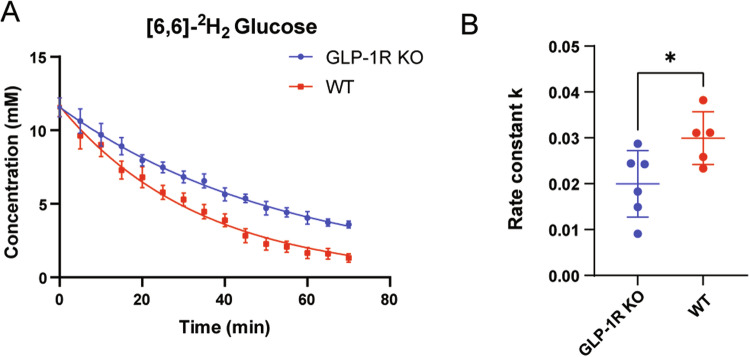
The metabolic rate of [6,6]-^2^H_2_ glucose in the WT and GLP-1R KO mouse brains. **A** Dynamics of deuterium-labeled glucose concentration. Each data point represents the mean ± SEM. **B** The rate constant (k) for [6,6]-^2^H_2_ glucose metabolism. The right side is a scatter plot, one dot per mouse, the wide bar is the mean, and the narrow bars are one standard deviation. The data represents mean ± SD, *p* = 0.0345, and statistical analysis was performed by unpaired two-tailed Student’s *t*-test. *N* = 6 for GLP-1R KO mice, *N* = 5 for WT mice.

According to previous studies on the relaxation time of deuterated compounds [23, 32], the T_1_ values of HDO and [6,6’]-^2^H_2_ glucose were set as 320 and 64 ms, respectively, during analysis. After relaxation correction, accurate concentration values of water and glucose could be obtained. We next fitted the glucose concentrations in the brains of each mouse after injection of [6,6’]-^2^H_2_ glucose. Glucose concentration in the mouse brain decreased rapidly after injection. It was 0 mM before injection and reached a maximum immediately after injection. However, due to the time constraints of the sequence, the rising process was not observed, thus only the falling phase of glucose was fitted in this experiment. The fitting results showed that the maximum concentration of glucose in GLP-1R KO mice was 11.56 ± 1.6 mM, whereas that in WT mice was 11.57 ± 1.4 mM. Thus, there was no significant difference between the two glucose concentrations at the maximum value. The glucose concentration showed a decreasing trend over time, but it decreased faster in WT mice (Fig. 2A).

We then calculated the rate of glucose metabolism within 0–70 min in both groups of mice. The results showed that, in GLP-1R KO mice, the glucose metabolism rate constant k was 0.01998 ± 0.07 mM/min, whereas in WT mice k was 0.02335 ± 0.057 mM/min. The results of our statistical analysis are shown in Fig. 2B; the metabolic constant of GLP-1R KO mice was significantly lower than that of WT mice (Unpaired *t*-test; *p* = 0.0345).

### Decreased brain glucose uptake in GLP-1R KO mice detected with [^18^F]-FDG-PET

**Fig. 3:**
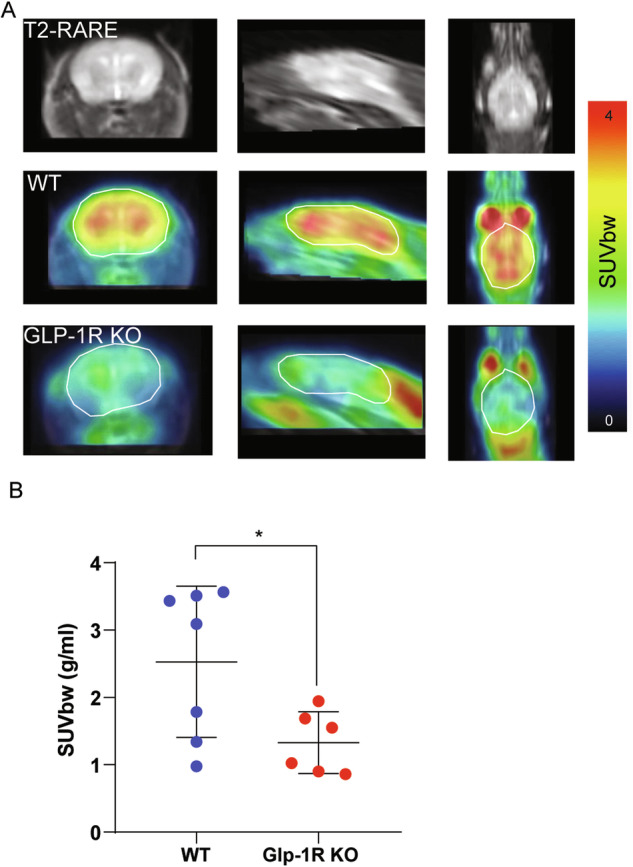
Glucose metabolism in the WT and GLP-1R KO mice brains measured with [^18^F]FDG-PET. **A** Representative PET/MRI images from coronal, sagittal, and axial views of WT and GLP-1R KO mice taken 45–60 min after 18 F-FDG injections. **B** Quantitative analysis of [^18^F]-FDG uptake in the whole brain. In this scatter plot, one dot per mouse, a wide bar is the mean, and narrow bars are one standard deviation. GPL-1R mice showed a significantly lower SUV_bw_ in the whole brain compared to WT mice. Data were presented as mean ± SD, *p* = 0.0314, statistical analysis was performed by unpaired two-tailed Student’s *t*-test with Welch’s test. *N* = 6 for GLP-1R KO mice, *N* = 7 for WT mice.

In clinical settings, [^18^F]-FDG-PET can be considered the gold standard for imaging glucose [50]. Here, [^18^F]-FDG-PET was used to evaluate whole-brain glucose metabolism in GLP1-1R KO and WT mice. For quantitative analysis [^18^F]-FDG uptake was measured in whole brain areas, and the standardized uptake value based on body weight (SUV_bw_) were calculated. All mice showed a physiological extracranial [^18^F]-FDG distribution with normal uptake in the Harderian glands, brown adipose tissue, myocardium, kidneys, gastrointestinal tract and urinary bladder. GPL-1R KO mice had a significantly lower SUV in the whole brain compared to WT mice (Unpaired *t*-test, *p* = 0.025, Fig. 3), which was consistent with the results of DMRS.

### Decreased whole-brain functional connectivity in GLP-1R KO mice

Previous studies have suggested a link between functional connectivity and the use of glucose in the brain [27, 51, 52]. Therefore, we also examined the whole-brain-wide functional connectivity of WT and GLP-1R KO mice.

**Fig. 4:**
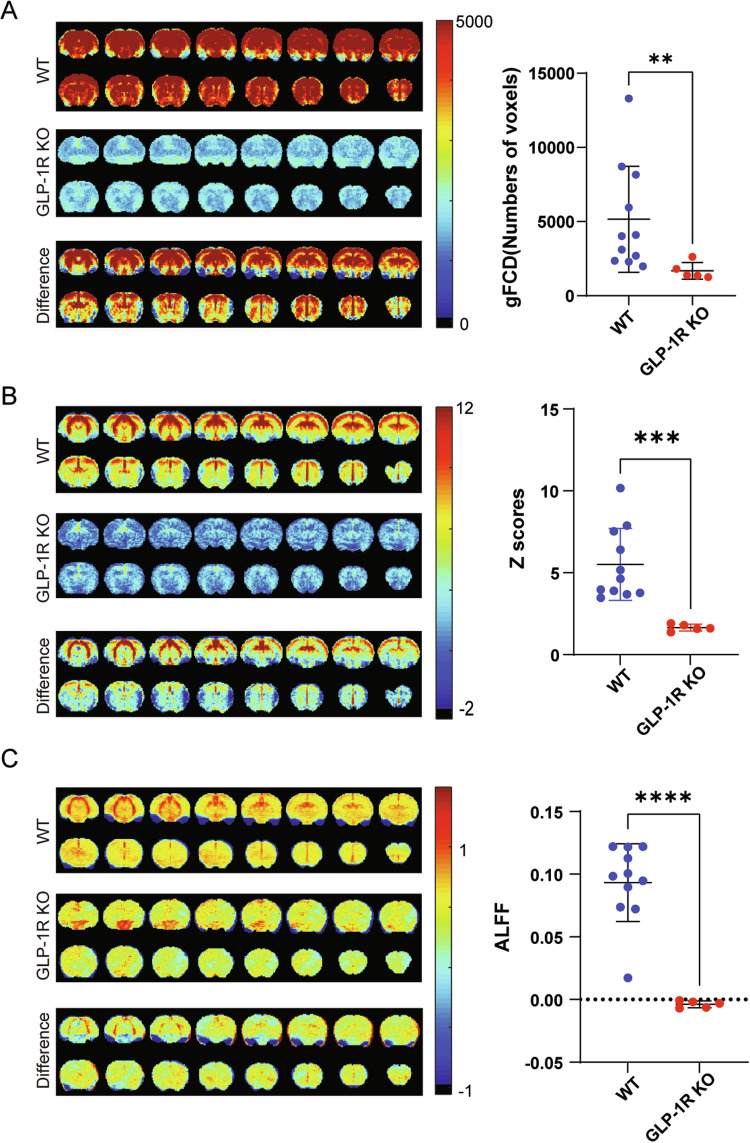
Comparison of functional connectivity averages across the whole brain in rs-fMRI between WT and GLP-1R KO mice. The left side is coronal images, 16 slices shown, an average of all mice in each group. The right side is a scatter plot, one dot per mouse, the wide bar is the mean, and the narrow bars are one standard deviation. **A** Functional connectivity density maps in the brain. **B** Whole-brain correlation maps in the brain. **C** The Amplitude of low-frequency fluctuations maps in the brain. The functional brain connectivity in GLP-1R KO mice was significantly lower than that in the WT group. *p* = 0.0032 for gFCD, statistical analysis was performed by Mann–Whitney test; *p* = 0.0002 for whole-brain correlation, *p* < 0.0001 for ALFF, statistical analysis was performed by unpaired two-tailed Student’s *t*-test with Welch’s test. Data are presented as mean ± SD, *N* = 5 for GLP-1R KO mice, *N* = 11 for WT mice.

Global functional connectivity density (gFCD) reflects the total number of connections between different voxels on a whole-brain scale [46]. GLP-1R KO mice showed lower gFCD than WT mice (Fig. 4A), suggesting that GLP-1R deletion resulted in reduced functional connectivity across the brain.

Whole-brain correlation is an indicator of the degree of interconnectivity and synergy between different brain regions [42]. GLP-1R KO mice showed lower whole-brain correlation than WT mice (Fig. 4B).

The amplitude of low-frequency fluctuation (ALFF) is a measure of low-frequency signals in the brain, which correlates with the strength of local neural activity [45]. The ALFF result in GLP-1R KO mice was significantly lower than that in WT mice (Fig. 4C).

### cAMP measurement

Since the GLP-1R KO mice showed reduced functional connectivity when measured across the whole brain, we further explored the impact of GLP-1 receptor knockout on downstream signaling pathways. GLP-1R predominantly signals through the Gα/cAMP pathway [7]. Thus, we examined the levels of cAMP in the whole brain tissues of WT and GLP-1R KO mice. The average level of cAMP in GLP-1R KO mice was slightly lower than that in WT mice, but there was no significant difference between the two groups (Fig. S3).

## Discussion

In this study, we applied DMRS, FDG-PET, and fMRI to explore the physiological role of GLP-1R in mouse brain glucose metabolism, metabolic homeostasis, and functional connectivity. We found that (1) GLP-1R KO mice exhibit impaired brain glucose metabolism to high doses of exogenous glucose; (2) GLP-1R KO mice have reduced functional connectivity when measured across the whole brain.

### Feasibility and validity of dynamic DMRS

In the dynamic DMRS study, we could accurately detect the dynamic changes of water and deuterated glucose, and we analyzed the differences in glucose metabolism between WT mice and GLP-1R KO mice by building a simple metabolic model, thus bypassing the difficulty in detecting glutamate and glutamine. While we drew blood for verification purposes from three mice (one representative mouse shown in Supplemental Fig. S4), the metabolic calculation was completed without these data, and thus future studies can feasibly obtain quantitative metabolic measurements through noninvasive DMRS.

### rs-fMRI correlates with the metabolic baseline

Brain activity and the physiological effects resulting from it are important and diverse, and require a large amount of energy from substances such as glucose. Some studies have suggested that brain regions with a high degree of functional connectivity are energy efficient and can minimize consumption of glucose [27, 51, 52]. Typically, higher functional connectivity implies more brain activity which requires more energy, this suggests a proportional relationship between functional connectivity and energy demand.

Previous studies have shown that higher glucose metabolism is associated with higher BOLD signal amplitudes, and that higher degrees of brain functional connectivity are associated with nonlinear increases in metabolism in healthy subjects by comparing PET and rs-fMRI. They compared rs-fMRI metrics, such as ALFF, functional connectivity, FCD, and regional homogeneity (which is similar to localized FCD), with the distribution of glucose uptake provided by FDG-PET. They have found that the spatial distribution of functional connectivity metrics correlates significantly with the spatial distribution of glucose uptake [51, 53].

We observed significantly decreased brain activity and functional connectivity with rs-fMRI. Reduced gFCD, which may indicate reduced information transfer between brain regions and disruption of functional networks. Reduced whole-brain correlation, which implies that connectivity and information transfer between different brain regions may have been affected. And finally, reduced ALFF, which implies a significant decrease in local activity. Together, these results reveal the impact of GLP-1R deletion on functional connectivity throughout the brain and further highlight the importance of GLP-1R in brain networks. Decreased functional connectivity may impede coordinated work and information transfer between brain regions, thereby interfering with normal metabolic regulatory processes [51].

### GLP-1R regulates brain glucose metabolism

The most prominent physiological effect of GLP-1 and GLP-1R is the regulation of insulin secretion in the pancreas to maintain postprandial glucose homeostasis, whereas outside the pancreas, GLP-1 and GLP-1R also regulate feeding and metabolism through the nervous system.

The inhibitory effect of GLP-1R agonists on food intake has been demonstrated in a number of species, including non-human primates [54] and humans [55]. Rodent studies [11, 56] have shown that central or peripheral administration of GLP-1R agonists reduces short-term food and water intake and lowers body weight. Similarly, peripherally administered GLP-1R agonists promote satiety, reduce energy intake, and lead to weight loss in healthy, diabetic, and obese individuals [5]. Recently, the development of GLP-1 agonists that show promise against obesity-associated disease named as Science’s 2023 Breakthrough (https://www.science.org/content/article/breakthrough-of-the-year-2023). Due to the different routes of administration and different GLP-1R analogs, GLP-1 and GLP-1R may be involved in different signaling pathways that directly or indirectly send signals to the central nervous system to inhibit ingestion and regulate eating behaviors by the nociceptors.

Previous studies showed that the basal blood glucose level of GLP-1R KO mice was upregulated over that of WT mice [57, 58]. Our work demonstrates that the response to exogenous glucose in the brain is also impaired in GLP-1R KO mice. This demonstrates that GLP-1R function is critical for normal brain metabolic function. Furthermore, this impairment is correlated with reduced functional connectivity in the brain. This correlation between glucose response and functional connectivity has been seen previously in healthy human subjects between the states of eyes open and eyes closed [27]. This suggests that GLP-1R mice may serve as a model of correlated metabolic-functional connectivity changes in the brain.

### Potential for research use of GLP-1R KO model

Correlated functional connectivity and metabolism in the brain is an exciting research area both because it creates possibilities for new medical imaging biomarkers [27], and also because the degree, or lack of, this correlation may be useful in detecting subtle changes in the brain [28]. Early studies indicated that brain regions of high FCD also had high glucose use, leading to a hypothesis that regions of high functional network activity, e.g. network hubs, had high energy demands [51, 52]. The correlation between functional connectivity and metabolism is sometimes preserved as the state of the brain changes, e.g. in healthy human volunteers, the eyes open state had correlated higher FCD, ALFF, whole-brain correlation, and glucose uptake than the eyes closed state [27] and awake mice had higher FCD [59], blood flow, and oxygen metabolism [60] than mice under dexmedetomidine anesthesia. These shifts in metabolism are often global across the brain, e.g., in a meta-analysis, the conditions of eyes closed versus open, sevoflurane and desflurane anesthesia, and comas of varying levels all demonstrated a global shift, whereas only congenitally blind versus sighted demonstrated localized changes [61].

A rodent model where the correlated functional connectivity and metabolism is observed would thus be valuable, as both noninvasive medical imaging and invasive neuroscience experiments could be performed. The GLP-1R KO mouse model provides such a model. In particular, whether pharmacological recovery of GLP-1R metabolic function or functional connectivity also restores the other is an interesting question both for medical imaging biomarkers and the development of such drugs. In addition, unlike studies in human volunteers, which have historically comprised the majority of fMRI research [62] the specific mechanisms that alter functional connectivity in GLP-1R KO mice versus WT mice can be investigated with the full toolset available to rodent neuroscience.

### Limitations and future work

Due to the smaller brains of mice versus rats and the 9.4 T vs. the 11.7 T field strength, we observed fewer peaks in the 2H spectrum as compared to De Feyter et al. [24] and some peaks such as lactate were inconsistently observed (Fig. S2). Because of this, we also increased the acquisition time for each spectrum to about 5 minutes. In the future, to improve spectral resolution and reduce scanning time, we and our collaborators have been preparing to develop a ^1^H-^2^H surface coil, which theoretically has higher sensitivity and resolution compared with the birdcage volumetric coil in this work. We are also testing more efficient shimming methods, as well as better spectrum processing methods with higher fitting accuracy, in the hope of obtaining higher-quality ^2^H-NMR spectra.

To explore the mechanism by which loss of GLP-1R caused impaired brain glucose metabolism, we examined the levels of cAMP in whole brain tissues. The results showed that the average level of cAMP in GLP-1R KO mice was slightly lower than that in WT mice, but there was no significant difference between the two groups. As an important downstream signaling molecule, cAMP is the target of regulation by many receptors after binding their ligands. Therefore, in GLP-1R KO mice, compensation mechanisms of other receptors may exist to balance the cAMP-mediated downstream signaling pathway to maintain normal survival and activities of the GLP-1R KO mice. Another reason may be that in the whole brain imaging studies in this manuscript, we focused on the resting state. In order to maintain consistency with the rest of our study, we did not attempt to alter neural activation prior to euthanizing mice in the cAMP detection experiment. Therefore, future work could investigate states other than the resting state. For example, at the cellular level, primary neuronal cells from GLP-1R KO and WT mice could be cultured, and the levels of cAMP and changes in downstream phosphorylation of PKA and CREB signaling pathway after GLP-1R agonist treatment could be tested.

## Conclusion

In conclusion, GLP-1R KO mice exhibit impaired brain glucose metabolism combined with reduced functional connectivity, a correlation previously observed in human studies that were unrelated to the GLP-1R gene. This suggests that the GLP-1R KO mouse model may serve as a model for correlated metabolic and functional connectivity loss. Our results are conducive to further understanding the regulatory effect of GLP-1R on glucose metabolism in the central nervous system, and provide a theoretical basis for the future development of GLP-1R-related drugs. In addition, our results help validate the recently developed techniques of DMRS and whole-brain functional connectivity as preclinical in vivo methods with good correspondence to existing PET methods.

## Supplementary information

Supporting information

## Data Availability

All data described in the manuscript are available upon reasonable request to the corresponding authors.

## Code availability

All codes described in the manuscript are available upon reasonable request to the corresponding authors.
