# Supplementary material for: Impaired brain glucose metabolism in glucagon-like peptide-1 receptor knockout mice
Source: Nutr Diabetes. 2024 Oct 10;14:86. doi: 10.1038/s41387-024-00343-w (PMC11466955; doi:10.1038/s41387-024-00343-w)
Supplement: Supplementary file 1 — Supporting information [file 41387_2024_343_MOESM1_ESM.docx]

**Supporting Information**

**Impaired brain glucose metabolism in glucagon-like peptide-1 receptor knockout mice**

Hui Li^1, 3#^, Yujiao Fang^1, 2, 3^, Da Wang^1,2^, Bowen Shi^1,2^, Garth J Thompson^1#^

**Affiliations**

1. iHuman Institute, ShanghaiTech University, Shanghai, China

2. School of Life Science and Technology, ShanghaiTech University, Shanghai, China

3. These authors contributed equally

**Contact information**

* Address correspondence to:

contact@garththompson.com, ShanghaiTech University, iHuman Institute, 393 Middle Huaxia Rd, Shanghai, 201210, China

lihui@shanghaitech.edu.cn, ShanghaiTech University, iHuman Institute, 393 Middle Huaxia Rd, Shanghai, 201210, China

**Table of Contents**

**Figure S1.** Depiction of the workflow of mouse MRI and PET scanning procedures.

**Figure S2.** Example spectrum of lactate in the brain of a WT mouse.

**Figure S3.** cAMP levels in WT and GLP-1R KO mice.

**Figure S4.** The spectra of [6,6’]-^2^H_2_ glucose in vitro deuterium glucose under 18.8T NMR system.

**Table S1.** The detailed information of F-test results and effect sizes of the data in this work.


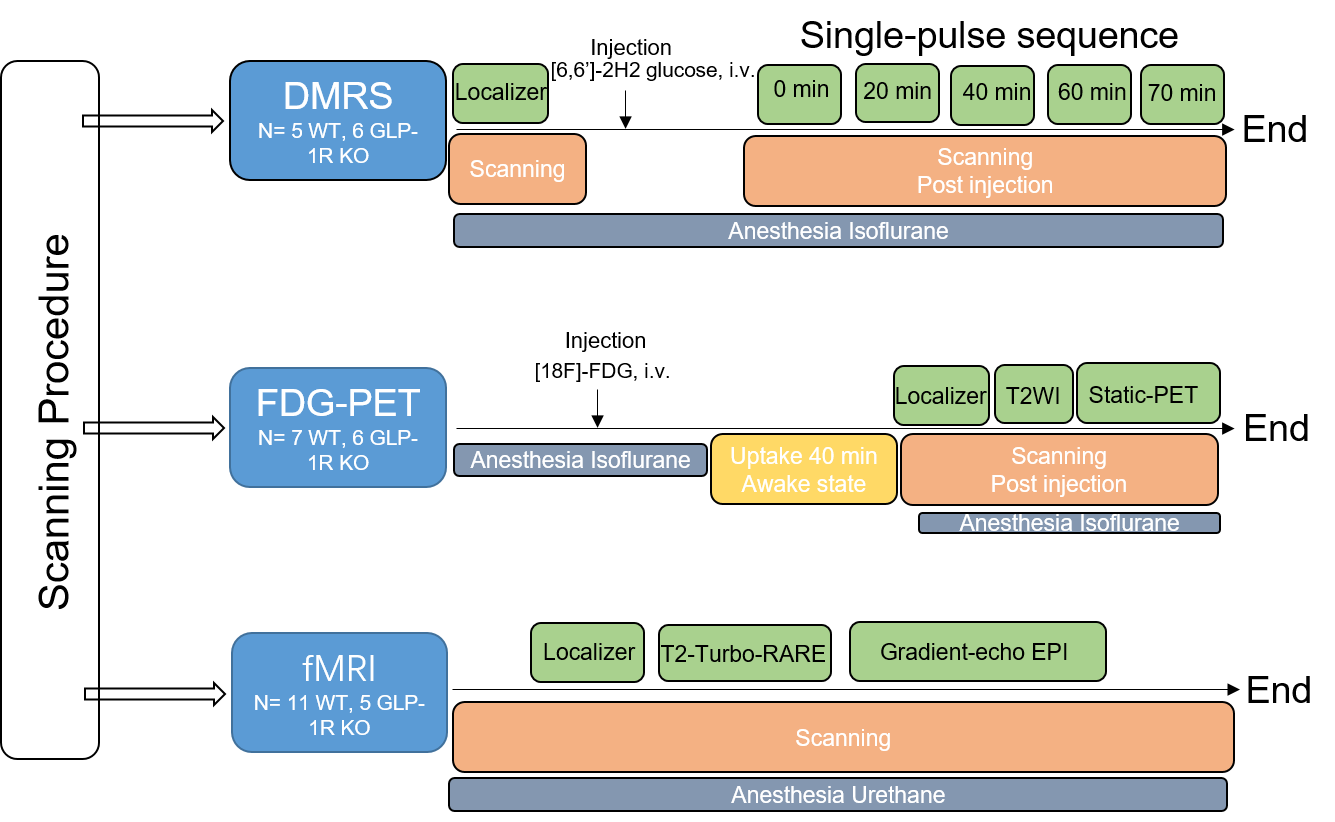


**Fig S1.** Depiction of the workflow of mouse MRI and PET scanning procedures.


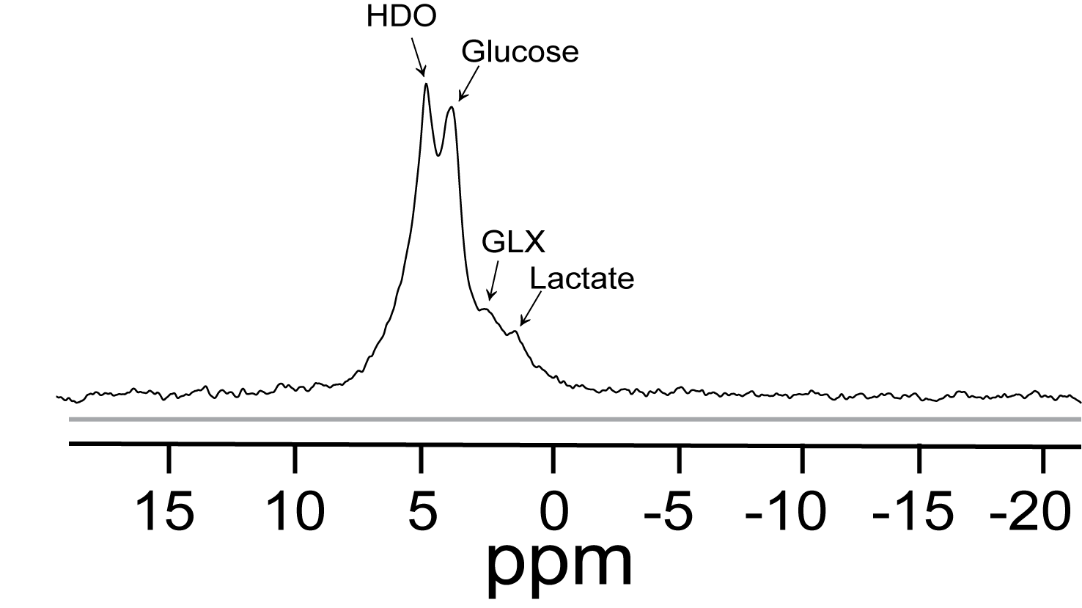


**Fig S2.** Example spectrum of lactate in the brain of a WT mouse. This spectrum was detected 20 minutes after infusion of [6,6’]-^2^H_2_ glucose. The peak of Glx was around 2.4 ppm, and the peak of lactate was around 1.3 ppm.


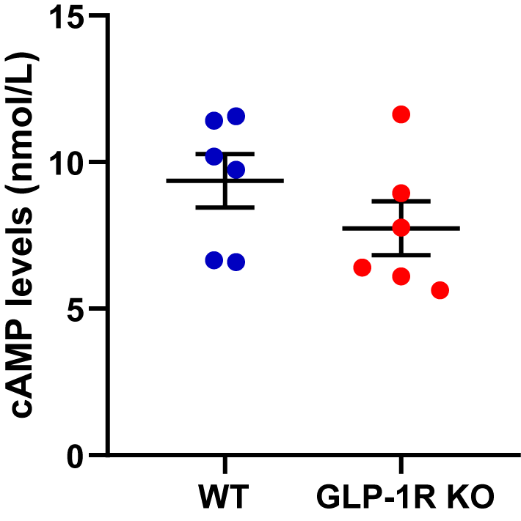


**Fig S3.** cAMP levels in WT and GLP-1R KO mice. Data are presented as mean ± SD, N = 6 for GLP-1R KO mice, N = 6 for WT mice.


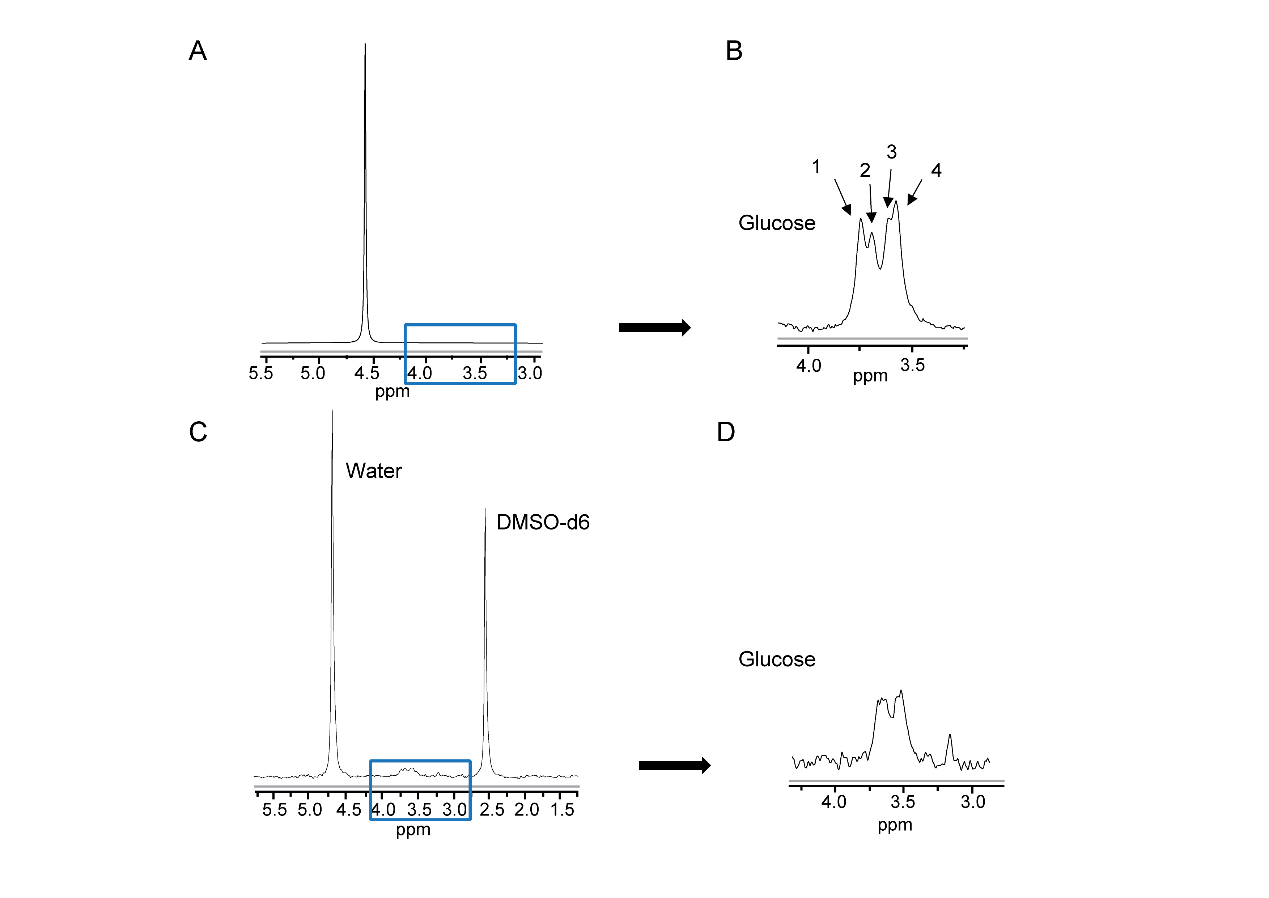
 **Fig S4.** The spectra of [6,6’]-^2^H_2_ glucose in vitro under 18.8T NMR system. One representative mouse shown. (A) and (B) represented a 40 mM aqueous solution of [6,6]-^2^H_2_ glucose. (C) and (D) represent plasma samples from WT mouse. (B) and (D) show magnified regions within the blue boxes in (A) and (C), respectively. 1, 2, 3, and 4 correspond to β^2^H6, α^2^H6, β^2^H6’, α^2^H6’.

|  | **Normal Distribution** | ***F*** | ***DFn*** | ***Dfd*** | ***F* variance** | **Test methods** | ***P* value** | **Effect size** |
| --- | --- | --- | --- | --- | --- | --- | --- | --- |
| **Fig 2B** | Yes | 1.592 | 5 | 4 | No | unpaired *t* (two tail) | 0.0345 | 1.7316 |
| **Fig 3B** | Yes | 5.990 | 6 | 5 | Yes | unpaired *t* (two tail) with Welch's test | 0.0314 | 1.0711 |
| **Fig 4A** | No | N/A | N/A | N/A | N/A | Mann-Whitney | 0.032 | 0.5785 |
| **Fig 4B** | Yes | 109.9 | 10 | 4 | Yes | unpaired *t* (two tail) with Welch's test | 0.0002 | 2.1747 |
| **Fig 4C** | Yes | 131.4 | 10 | 4 | Yes | unpaired *t* (two tail) with Welch's test | <0.0001 | 3.1367 |

**Table S1.** The detailed information of F-test results and effect sizes of the data in this work.
